# Seroprevalence of Brucellosis in Haryana, India: A Study Using Rose Bengal Plate Test and Enzyme-Linked Immunosorbent Assay

**DOI:** 10.3390/pathogens14040373

**Published:** 2025-04-10

**Authors:** Dinesh Mittal, Kushal Grakh, Manesh Kumar, Punit Jhandai, Swati Dahiya, Renu Gupta, Ramesh Kumar, Anand Prakash, Pankaj Kumar, Pallavi Moudgil, Rajesh Khurana, Naresh Jindal

**Affiliations:** 1 Department of Veterinary Public Health and Epidemiology, Lala Lajpat Rai University of Veterinary and Animal Sciences, Hisar 125004, Haryana, India; kushalgrakh24@gmail.com (K.G.); vetmanesh@gmail.com (M.K.); punit41521@gmail.com (P.J.); drenugupta@gmail.com (R.G.); rameshkdr@gmail.com (R.K.); anandprakash@luvas.edu.in (A.P.); drpankaj42@gmail.com (P.K.); pallavi.moudgil@luvas.edu.in (P.M.); khurana.rajesh846@gmail.com (R.K.); nareshjindal1@gmail.com (N.J.); 2 Department of Veterinary Microbiology, Lala Lajpat Rai University of Veterinary and Animal Sciences, Hisar 125004, Haryana, India; swatidahiya@luvas.edu.in

**Keywords:** brucellosis, bovine, i-ELISA, Haryana, India, RBPT

## Abstract

Brucellosis, a contagious reproductive disease of livestock, has a significant economic impact in terms of abortions and stillbirths and has zoonotic importance. A study was conducted to estimate the seroprevalence of brucellosis in a bovine population in Haryana state, India. This study was carried out on 4325 bovine serum samples (Cattle: 2151, Buffalo: 2174) using the Rose Bengal Plate Test (RBPT) and indirect enzyme-linked immunosorbent assay (i-ELISA). The seroprevalence, estimated individually by the RBPT and i-ELISA, was found to be 6.86% (95% CI: 6.11–7.62) and 6.05% (95% CI: 5.34–6.76), respectively. In total, 258 out of 4325 (5.96%; 95% CI: 5.25–6.67) samples were found to be positive by both assays. The prevalence was found to be significantly higher in the cattle population (7.58%) as compared to buffalo (4.37%) (Chi-square = 19, *p* < 0.001). Seroprevalence was highest in the agroclimatic zone I (8.73%), followed by zone II (7.33%) and zone III (1.45%) (Chi-square = 76.27, *p* < 0.001).

## 1. Introduction

Brucellosis, also known as Bang’s disease, is primarily a contagious reproductive disease of animals with significant economic impact. It is caused by organisms of the genus *Brucella* and is known to infect both domestic and wild terrestrial animals, with few cases reported in marine animals [1]. The disease is initially mild, with few or no symptoms in the infected animal, which may later develop into the most devastating disease in terms of abortions (30% to 80%) and stillbirths. It is also considered hazardous to humans due to its zoonotic nature [2,3,4]. Other prominent effects of the disease include the birth of weak or vulnerable offspring, retention of fetal membranes, infection of the membranes, secondary metritis, and decreased lactation [5]. The economic impact may be direct in terms of a reduction in milk yield and higher mortality, or indirect through the cost of vaccination and culling. The disease in cattle and buffalo accounts for 95.6% of the total losses due to brucellosis in livestock populations, with an estimated economic median loss of USD 3.4 billion, including a loss of USD 6.8 per cattle and USD 18.2 per buffalo in India [6].

Numerous nations, including India, have developed control strategies to eradicate brucellosis in bovine populations. The Government of India (GOI) started the National Animal Disease Control Program in 2019 to combat brucellosis and foot and mouth disease (FMD). The *Brucella* vaccine S19 will be provided to all 36 million female calves aged 4–8 months [7,8,9,10].

Since almost all *Brucella* species are harmful to human and animal health, it is imperative to detect the pathogen in the early stages of the disease. The timely implementation of appropriate public health programs depends on rapid laboratory diagnostics for pathogen identification. Although culture-based isolation is considered the “gold standard” for the diagnosis of brucellosis, the major constraint is the requirement for certified BSL-3 facilities for processing [11].

Although some isolated studies in the state of Haryana have reported varying seroprevalence estimates across different districts, these studies covered a smaller number of districts, with small sample sizes, and some were limited to a single species or sex, leaving a gap to be addressed. The current study was designed to use a systematic approach for estimating the seroprevalence of brucellosis using the Rose Bengal Plate Test (RBPT) and the indirect enzyme-linked immunosorbent assay (i-ELISA).

## 2. Materials and Methods

### 2.1. Study Area and Sampling

The present study was performed in 22 districts of the northern state of India, Haryana, spanning between 27°39′ and 30°35′ N latitude and 74°28′ and 77°36’ E longitude. According to the Livestock census 2019, the state is home to 1.93 million cattle and 4.34 million buffaloes. Based on the ecology and cropping patterns, the ICAR-Agricultural Technology Application Research Institute, Jodhpur (India), has classified the state into three distinct zones (I, II, and III). Zone I (eight districts) comprises Ambala, Karnal, Kaithal, Kurukshetra, Panchkula, Panipat, Sonipat, and Yamunanagar; zone II (seven districts) consists of Fatehabad, Faridabad, Hisar, Jind, Rohtak, Sirsa, and Palwal; and zone III (seven districts) includes Bhiwani, Charkhi Dadri, Mahendragarh, Rewari, Jhajjar, Gurugram, and Mewat [12].

The serum samples were obtained from the jurisdictions of Government Veterinary Hospitals (GVHs) throughout the state under the Livestock Health and Disease Control Program for foot and mouth disease surveillance. In total, 4325 bovine serum samples (Cattle and Buffalo) were collected: 1580 from zone I, 1365 from zone II, and 1380 from zone III.

### 2.2. Sample Processing

All the serum samples were processed for the detection of antibodies using the Rose Bengal Precipitation Test (RBPT) and ELISA. As this study was conducted under an active surveillance program (Outreach Program on Zoonotic Diseases; OPZD), the sample size estimation was not undertaken. The Veterinary Surgeon (VS) of the respective GVH in each village, under the jurisdiction of the respective district, collected the samples randomly from their respective fields. The animal owners were informed, and consent was obtained before sample collection. Additionally, age, sex, species, and breed data were also recorded from each household. The samples were stored at −20 °C and were thawed at 37 °C before testing. All tests were performed in the Disease Investigation (DI) Laboratory in the Department of Veterinary Public Health and Epidemiology, LUVAS, Hisar, Haryana (India).

### 2.3. Rose Bengal Plate Test (RBPT)

All the samples were subjected to the RBPT using the *B*. *abortus* S99 strain (IVRI, Izzatnagar, Bareilly, India) to detect *Brucella* antibodies. Briefly, on a glass plate, equal volumes (30 μL) of antigen and test serum were mixed thoroughly using a toothpick, and the mixture was gently agitated for 3–4 min at room temperature. Any agglutination (observed as spots, flakes, or dotted particles) was considered a positive reaction [13]. In parallel, positive and negative control serum samples available at the DI Laboratory, VPHE, LUVAS, were used for quality control.

### 2.4. Indirect Enzyme-Linked Immunosorbent Assay (i-ELISA)

All serum samples were subjected to an indirect ELISA (i-ELISA) using the PrioCHECK^®^ BRUCELLA Ab 2.0 ELISA (Thermo Fisher Scientific, Waltham, MA, USA) to detect IgG antibodies against *Brucella* spp., as per the manufacturer’s protocol. The results were interpreted using a multimode microplate absorbance reader at 450 nm. The OD450 of all samples was expressed as percent positivity (PP) relative to the mean OD450 of the Positive Control.

PP = (OD_450 test sample_/mean OD_450 Positive Control_) × 100

The baseline cut-off (PP) was set at 40%, and samples with a PP below this cut-off were considered negative.

### 2.5. Statistical Analysis

To correlate the categorical variables with disease prevalence, a bivariate analysis was conducted. A generalized linear model was fitted to identify associated risk factors for brucellosis among livestock. The confidence intervals (CIs) were calculated using multiple variables, including categorical variables such as age, sex, location of sample collection, and bovine species. Serological results were presented independently for both the RBPT and i-ELISA, and the overall prevalence was reported separately and considered as the final/true positive. All the data were analyzed using R Studio (version 2023.12.1+402) and Microsoft Excel 2021 (version 2311), and all statistical conclusions were deemed significant with a *p*-value of less than 0.05 at a minimum confidence level of 95%.

## 3. Results

A total of 4325 samples were used for the diagnosis of brucellosis by the RBPT and i-ELISA. The prevalence of brucellosis among the bovine population was 6.86% (296/4325) (95% CI: 6.11–7.62) using the RBPT test. Among the zones, zone I exhibited the highest seropositivity at 10.70% (169/1580), followed by zone II at 7.25% (99/1365) and zone III at 2.10% (29/1380) (Table 1). The seropositivity was significantly higher in zone I and zone II compared to zone III (*p* < 0.001). The analysis concerning species revealed a significantly higher (*p* < 0.001) seropositivity of 9.30% in cattle, as compared to buffaloes (4.46%) (Table 1). The seroprevalence was higher in the older age group (>5 years; 7.68%), followed by the 1–5 years of age group (6.42%), and the less than one year group (6.02%), though none of these were significantly associated. The RBPT revealed a slightly higher seroprevalence among male animals (5.50%), although this was not significantly associated (Table 1).

The seroprevalence measured using i-ELISA was 6.05% (262/4325) (95% CI: 5.34–6.76). Similar to the trend in the RBPT, zone I exhibited the highest seropositivity at 8.80% (139/1580), followed by zone II at 7.47% (102/1365), both of which were also significantly associated (*p* < 0.001), as compared to zone III at 1.52% (21/1380) (Table 1). A significantly higher (*p* < 0.001) seropositivity of 7.72% in cattle was observed compared to buffaloes (4.42%) (Table 1). The seroprevalence was slightly higher in the older age group (>5 years; 6.47%), followed by the 1–5 years of age group (5.82%), and the less than one year age group (6.02%). The iELISA revealed a slightly higher (non-significant) seroprevalence among female animals (6.08%) (Table 1).

**Figure 1:**
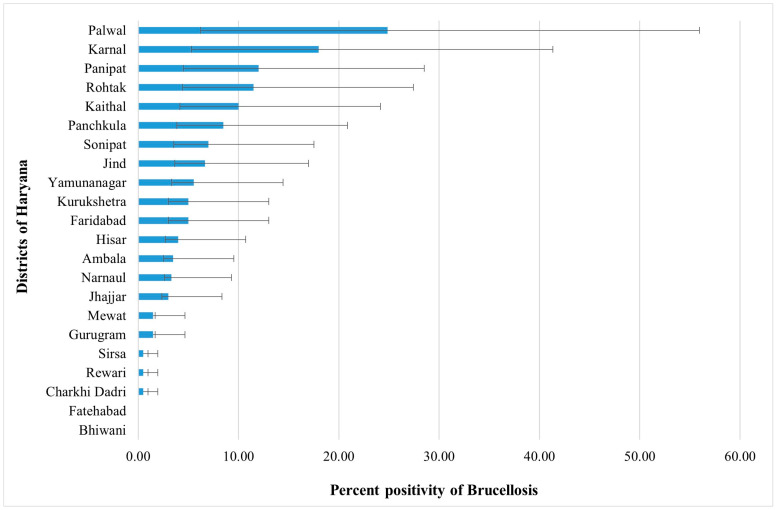
The percent positivity of brucellosis in serum samples for different districts in the state of Haryana. (The error bars represent the 95% CI).

Overall seroprevalence was calculated as the number of positive samples from both diagnostic assays, viz., the RBPT and i-ELISA. The overall prevalence was 5.97% (95% CI: 5.25–6.67) and was taken as the final seroprevalence value for further reporting and analysis. Similar to trend in the RBPT and i-ELISA among the geographic zones, zone I and zone II exhibited a significantly higher (*p* < 0.001) seropositivity at 8.73% (138/1580) and 7.33% (100/1365), respectively, as compared to zone III at 1.45% (20/1380) (Table 1). A significantly higher (Chi-square = 19, *p* < 0.001) seropositivity of 7.58% in cattle was recorded as compared to buffaloes (4.37%) (Table 1). The seroprevalence was slightly higher in the older age group (>5 years; 6.40%), followed by the 1–5 years of age group (5.71%), and the less than one year age group (6.02%). The i-ELISA revealed a slightly higher (non-significant) seroprevalence among female animals (6.08%) (Table 1). Moreover, brucellosis seroprevalence in the male bovine population of the state was found to be 5.50% (11/200). Of the two-hundred male bovine serum samples tested, six out of six were positive for the age group of less than one year, five out of one-hundred and forty-nine were positive for the age group of 1–5 years, and none of the forty-five samples tested positive for the age group of more than 5 years. The highest prevalence in the bovine population was observed in Palwal district (24.32%), followed by Karnal, Panipat, and other districts (Figure 1). Bhiwani and Fatehabad were the only two districts where no overall positivity was observed. The results of the association between different variables and the brucellosis prevalence are depicted in Table 1.

The deviance residuals ranged from −2.53 to 0.41 (median 0.31), indicating a reasonable fit of the model. The dispersion parameter for the binomial family was assumed to be 1. Among the predictors, species (cattle) had a significant negative association with the outcome (β = −0.56, *p* < 0.001), indicating that cattle were associated with reduced odds of the outcome compared to the reference category (buffalo), i.e., cattle are significantly less likely to test negative as compared to buffalo. The effect of age (less than 1 year) was not statistically significant (β = 0.14, *p* = 0.304), suggesting the absence of a significant association between this age group and the outcome compared to the reference category (age > 1 year). Similarly, the effect of sex (male) was not statistically significant (β = 0.05, *p* = 0.871). A kappa value of 0.914 indicated an almost perfect agreement between the two tests, i.e., the RBPT and i-ELISA.

## 4. Discussion

A high prevalence of brucellosis is observed worldwide, including in India, and it is the main cause of late-term abortions in both large and small ruminants, particularly cattle, buffaloes, sheep, and goats. The disease is typically identified using culture procedures and serological assays like the RBPT, ELISA, CFT, and SAT. While the isolation of the causative organism (*Brucella* spp.) is regarded as the gold standard, the requirement for BSL-3 facilities and its zoonotic nature constrain its applicability. The isolation of *Brucella* was not attempted in the current study. However, an earlier study conducted by Chand and Chhabra [14] in Haryana, India isolated *Brucella* species from aborted fetuses, indicating the active circulation of the pathogen in dairy farms in Haryana. In this study, the seroprevalence of brucellosis among bovine serum samples (n = 4325) was assessed using two diagnostic tests: the RBPT and i-ELISA. The findings revealed a slightly higher seropositivity using the RBPT (6.86%) compared to i-ELISA (6.05%). The overall seroprevalence (positive for both RBPT and i-ELISA) was 5.97% (95% CI: 5.25–6.67) and is comparable to earlier reports from different parts of India and neighboring countries. However, the seroprevalence recorded in the current study is lower than the findings of Jaismon et al. [15], who, in a meta-analysis, reported pooled prevalence estimates of brucellosis at 16.6% (95% CI: 13.0, 21.1) in cattle, 14.2% (95% CI: 8.9, 21.8) in buffaloes, and 15.1% (95% CI: 12.0, 18.8) in bovines (combined) using different diagnostic tests and sample types (milk and serum).

The study by Khurana et al. [16] in 11 districts of Haryana reported a slightly lower seroprevalence of 5.45% in cattle and 6.6% in buffaloes using the RBPT, suggesting the continued endemicity of brucellosis in the region. However, the seroprevalence observed in other states of India ranged from 5.06 to 9.66% in Rajasthan [17,18], 6.66% in Tamil Nadu [19], 6.4% in Meghalaya [20], and 9.66% in Gujarat [21]. The earlier studies reported a lower occurrence of brucellosis in buffaloes, ranging from 1.9 to 4.2% using the RBPT [22,23], and higher herd prevalence, ranging from 12.73% to 47.6% in the state of Haryana, India and the neighboring country, Pakistan [24,25,26,27,28]. The wide variation in seroprevalence may be due to differences in sample sizes, sampling techniques, selection of animal farms, animal density at the farms, management practices (traditional/scientific/mixed farming), and religious practices (particularly in cattle) in these regions.

The slightly higher prevalence detected by the RBPT (6.84%) compared to i-ELISA (6.05%) may be attributed to the higher sensitivity of the RBPT in detecting early or recent infections. The i-ELISA offers better specificity, particularly for chronic cases, emphasizing the value of using both tests in tandem for robust prevalence estimates. The generalized linear model validated these findings, with the deviance residuals indicating a reasonable model fit. The negative association between species (cattle) and a negative test outcome reinforces the higher susceptibility of cattle compared to buffalo. A high diagnostic accuracy for the RBPT (~95.9%) has been observed for brucellosis diagnosis across studies [29]. In another human-centric study, the RBPT test was found to be highly specific (95.7%) [30]. Contrarily, the indirect ELISA (i-ELISA) can also detect brucellosis in milk and serum samples, with a 99.1% specificity [31]. The current study did not assess the specificity of i-ELISA, but the selection of this serological technique is logical based on the high specificity and sensitivity of the test [32]. The use of the i-ELISA and RBPT in combination is endorsed by the OIE, which recommends the use of at least two tests at a time for better brucellosis screening of the bovine population [33]. Although the RBPT is a simple, quick, and sensitive test for detecting bovine brucellosis, it may yield non-specific reactions with other Gram-negative bacteria that share antigenic similarities with Brucella [34]. The lipopolysaccharide molecules, possessing similar antigenic epitopes, can cross-react with other Gram-negative bacteria like Yersinia enterocolitica O:9, *Escherichia coli* O116 and O157, *Salmonella enterica* serovars, and *Vibrio cholerae*. To avoid non-specific reactions, it is recommended that serum samples be further tested using other reliable and specific methods, such as the ELISA or CFT, to ensure a definitive diagnosis. The specificity of CFT is excellent, but its sensitivity is lower compared to the RBPT and I-ELISA. Additionally, CFT requires a more specialized laboratory [35]. For bovine brucellosis, the ELISA has a higher detection capability [36] and can help avoid non-specific reactions in RBPT-positive sera [37]. However, in the later stages of brucellosis, non-agglutinating antibodies become more prevalent than agglutinating antibodies, which may result in missed detections. Combining the RBPT with ELISA ensures the identification of all Brucella-positive reactors. It is not possible to detect all Brucella reactors with a single agglutination test [38]. Therefore, combining the RBPT with ELISA is recommended, as it helps prevent unnecessary slaughter or segregation due to false positives, as well as the risk of brucellosis spread from retaining infected animals due to false negatives.

The overall and individual seroprevalence by the RBPT and i-ELISA were significantly higher in zone I (8.73%) and zone II (7.33%) compared to zone III (1.45%) (Table 1). This significant regional variation may be potentially driven by differences in livestock density, management practices, and biosecurity measures in this part of India. Muthiah et al. [39] reported a higher (non-significant) seroprevalence in zone II than in zones I and III of Haryana, possibly due to a smaller sample size from a single species and sex, i.e., female buffalo. In 2012, Khurana et al. [16] reported a higher prevalence among zone II districts, without including zone I districts for comparison. The different sample sizes, sampling bias, and confounding variables may have contributed to the variations in results. The current study was conducted using a larger and more consistent sample size, and calculations were performed using stratified random sampling to avoid sampling bias. This approach helped in the comparative analysis and avoided the underrepresentation of smaller districts, as different districts of Haryana have varying bovine population densities. Districts like Bhiwani and Fatehabad exhibited no overall seropositivity, which might be due to effective disease control measures, differences in sampling intensity, or lower exposure risk. Vaccination against brucellosis was not practiced in Haryana at the time of sample collection. It appears that the variations in seropositivity across zones may be due to differences in livestock population, rearing practices, and pasture areas. As zones I and II have abundant land cover, pasture areas, and higher livestock populations, these conditions may facilitate interactions between animals, enabling transmission [39]. The conducive environment may foster interactions between different species, facilitating the transmission of diseases. The establishment of new dairy farms, an increase in animal density, and the replacement of old animals with new dairy animals for better livelihood may have accelerated the transmission of brucellosis due to the introduction of already infected animals by farmers. Feedback from farmers indicated that, most of the time, animals are purchased and introduced into the farm without brucellosis testing or quarantine practices in this part of India.

The strong agreement (kappa = 0.914) among the serological tests (RBPT and i-ELISA) observed in the current study is in line with earlier reports [28,39]. A higher seroprevalence was observed in cattle as compared to buffaloes, similar to earlier reports from Rajasthan [18] and Haryana [16,28]. Cattle are more prone to brucellosis infection than buffaloes [15,40,41] in India due to specific management practices for cattle, intensive dairy systems with close contact, religious sentiments involving cattle (no slaughter, keeping cattle until death), and stray cattle facilitating disease transmission [42]. Buffaloes, often reared under extensive management conditions, may experience a lower exposure to the pathogen, thereby reducing the risk of infection. Hence, a higher disease prevalence is observed in cattle compared to buffaloes [28,43,44]. The observed higher brucellosis prevalence in cattle may also be due to widespread cattle movement and mixing with other animal herds at common grazing/water points. The brucellosis prevalence was slightly higher in animals over five years of age (6.40%) compared to younger groups, which might be because older animals have prolonged contact with infected animals over time during breeding, parturition, milking, etc. [22]. Although bovines of all age groups are affected by brucellosis, susceptibility is greatly influenced by age, sex, and reproductive status. However, it is the sexual maturity that makes the animal more prone to brucellosis [45], leading to a higher brucellosis seroprevalence in older age groups [23,28].

No significant association between the sex of the animal and brucellosis was observed in the current study, although there are reports indicating a higher seroprevalence in female animals as compared to male animals [23,46], possibly due to the introduction of unscreened females into the herd. Brucellosis affects both males and females equally, but the disease’s clinical manifestations are primarily seen in females (e.g., abortion and retention of placenta), whereas males typically experience orchitis [10].

This study’s observations indicate that brucellosis is present in bovine bulls, affecting both young and adult animals. The bulls may have contracted the infection from the contaminated environment [47], as brucellosis is endemic in Haryana, with recent reports indicating a high prevalence of the disease in the state [39].

## 5. Conclusions

The present study revealed a seropositivity rate of 5.96% for brucellosis in the bovine population of Haryana. The high prevalence of the disease indicates a significant risk of transmission of this zoonosis, posing a serious threat to both public health and livestock productivity. The high occurrence of brucellosis in older age groups, particularly those in the milch category, is a significant public health concern due to the potential excretion of the zoonotic *Brucella* spp. pathogens in milk. As the current study was conducted across the entire state of Haryana, its findings contribute to a better understanding of the distribution and status of brucellosis in the region. Additionally, the generated epidemiological data will serve as baseline information for developing effective prevention and control strategies for this important zoonotic disease, both in Haryana and across India. Initial screenings for bovine brucellosis may be carried out using RBPT, as it is a simple, quick, sensitive, and economical field test for detecting bovine brucellosis. The combined use of the RBPT and i-ELISA is recommended, given their diagnostic efficacy in identifying all *Brucella*-positive reactors in a herd, as it minimizes false positive and false negative outcomes.

## 6. Implications and Recommendations

As brucellosis is an important disease due to its economic importance and zoonotic nature, the Government of India has already initiated a control and prevention program both at both the state and national levels. To raise public awareness, effective communication methods, such as mass media, information and communication technology, and extensive education campaigns about the disease’s risk factors, economic importance, and zoonotic nature, are necessary, particularly in high-risk areas. Veterinary practitioners and livestock owners must be actively engaged in these efforts. There is also a need for the continuous sero-surveillance of the disease to assess the impact of control and prevention programs. As brucellosis is a major global public health concern, a combined effort using the “One Health” approach is essential to curtail the disease.

## Figures and Tables

**Table 1:** Chi-Square test results for association between brucellosis result and species, age, and sex.

	Variable	Tested Samples	Brucellosis								
			Positive by ELISA (%)	95% CI	Chi-Square (*p*-Value)	Positive by RBPT (%)	95% CI	Chi-Square (*p*-Value)	Overall Positive (%)	95% CI	Chi-Square (*p*-Value)
Overall		4325	262 (6.05)	5.34–6.76	-	297 (6.86)	6.11–7.62	-	258 (5.96)	5.25–6.67	-
Agroclimatic zone	Zone I	1580	139 (8.80)	7.40–10.19	75.53 (*p* < 0.001)	169 (10.70)	9.17–12.22	85.54 (*p* < 0.001)	138 (8.73)	7.34–10.13	76.27 (0.001) *
	Zone II	1365	102 (7.47)	6.08–8.87		99 (7.25)	5.08–8.63		100 (7.33)	5.94–8.71	
	Zone III	1380	21 (1.52)	0.88–2.17		29 (2.10)	1.34–2.86		20 (1.45)	0.82–2.08	
Species	Cattle	2151	166 (7.72)	6.59–8.85	20.70 (*p* < 0.001)	200 (9.30)	8.07–10.53	39.54 (*p* < 0.001)	163 (7.58)	6.46–8.70	19 (0.001) *
	Buffalo	2174	96 (4.42)	3.55–5.28		97 (4.46)	3.59–5.32		95 (4.37)	3.51–5.23	
Age	Less than 1 year	83	5 (6.02)	0.91–11.14	0.722 (*p* > 0.05)	5 (6.02)	0.91–11.4	2.56 (*p* > 0.05)	5 (6.02)	0.91–11.14	0.846 (0.655)
	1–5 year	2680	156 (5.82)	4.93–6.71		172 (6.42)	5.49–7.35		153 (5.70)	4.83–6.59	
	>5 year	1562	101 (6.47)	5.25–7.69		120 (7.68)	6.36–9.00		100 (6.40)	5.19–7.62	
Sex	Male	200	11 (5.50)	2.34–8.66	0.713 (*p* > 0.05)	11 (5.50)	2.34–8.66	0.613 (*p* > 0.05)	11 (5.5)	2.34–8.66	0.081 (0.776)
	Female	4125	251 (6.08)	5.36–6.81		189 (4.58)	3.94–5.22		247 (5.98)	5.26–6.71	

* Indicates the *p* values of variables which are significant.

## Data Availability

All the supporting information has been provided in the text itself. Any other supporting files, if requested, can be provided by the corresponding author.
